# Derazantinib enhances gemcitabine efficacy in PDAC by attenuating the NF-κB and MAPK pathways to suppress MUC5AC expression

**DOI:** 10.1007/s12032-025-03222-1

**Published:** 2025-12-30

**Authors:** Wenkai Ye, Yiyun Huang, Lue Hong, Yan Ma, Junnan Huang, Fangyue Xu, Fang Han, Yaju Qiu, Zhimiao Zou, Yuhua Zhang, Xia Li

**Affiliations:** 1 The Second School of Clinical Medicine, Zhejiang Chinese Medical University, Hangzhou, 310053 Zhejiang China; 2 Hangzhou Institute of Medicine (HIM), Zhejiang Cancer Hospital, Chinese Academy of Sciences, Hangzhou, 310022 Zhejiang China; 3 The First School of Clinical Medicine, Zhejiang Chinese Medical University, Hangzhou, 310053 China; 4 Zhejiang Cancer Research Institute, Zhejiang Cancer Hospital, Hangzhou, 310022 Zhejiang China

**Keywords:** PDAC, FGFR, Derazantinib, MUC5AC, Gemcitabine resistance

## Abstract

Pancreatic ductal adenocarcinoma (PDAC) is usually treated with gemcitabine (GEM)-based chemotherapy. However, resistance to GEM develops frequently. Aberrant fibroblast growth factor receptors (FGFR) signaling in PDAC is linked to advanced tumor stages and poor prognosis, making it a potential therapeutic target. This study aimed to explore whether inhibition of FGFR by derazantinib could reduce the resistance to GEM in PDAC. Human AsPC-1 and BxPC-3 PDAC cell lines were screened against a panel of FDA-approved compounds to identify the potential drug. Gem-resistant cell lines were subsequently utilized to validate the efficacy of derazantinib. The synergistic interaction between derazantinib and GEM was confirmed through combination-index analysis, clonogenic assays, and apoptosis assays. With RNA-seq, immunohistochemistry, Western blotting, and animal experiments, the effects of derazantinib on the malignant behaviors, signaling pathways in GEM-resistant PDAC cells and tumors were examined. Treatment with derazantinib and GEM synergistically inhibited the malignant behaviors of GEM-resistant PDAC cells and tumor growth by downregulating FGFR2 and FGFR3 expression. RNA-seq revealed upregulated MUC5AC expression in GEM-resistant PDAC, which was attenuated by derazantinib through inhibiting the MAPK and NF-κB signaling. Furthermore, higher levels of FGFR2 and FGFR3 expression were associated with worse survival of PDAC patients and negatively correlated with tumor differentiation. Moreover, the combination of derazantinib and GEM significantly inhibited the growth of GEM-resistant PDAC tumors in vivo. The data highlighted that higher levels of FGFR2, FGFR3, and MUC5AC expression promoted the progression and resistance to GEM in PDAC. Derazantinib treatment enhanced the sensitivity to GEM by attenuating the NF-κB and MAPK pathways to inhibit MUC5AC expression. Therefore, derazantinib may be a promising chemotherapeutic adjuvant for treating PDAC, particularly for patients with GEM resistance.

**Supplementary Information:**

The online version contains supplementary material available at 10.1007/s12032-025-03222-1.

## Introduction

Pancreatic ductal adenocarcinoma (PDAC) is one of the most lethal malignancies of the digestive system, characterized by highly aggressive biological behavior and an extremely poor clinical prognosis [1]. Globally, both the incidence and mortality rates of PDAC have steadily increased over the past decades [2, 3]. Over the past 40 years, despite advances in surgical techniques and systemic therapies improvements in long-term survival remain limited. The overall 5-year survival rate of PDAC is merely 8%, making it the solid tumor with the worst prognosis [4]. The development of PDAC is insidious and highly aggressive. When diagnosis, there are about 15%−20% of patients, who are suitable for surgical resection, while the majority of patients have already the tumor locally advanced or distant metastasis [1, 5]. For these patients, systemic chemotherapy based on gemcitabine (GEM) remains the first-line treatment option [6].

Since its approval in 1997, GEM has remained a cornerstone of PDAC therapy, either as monotherapy or as part of combination regimens such as GEM plus nab-paclitaxel in adjuvant or neoadjuvant therapy [7, 8]. However, the therapeutic efficacy of GEM is limited due to its short plasma half-life, poor targeting, and the chemoresistance of PDAC cells [9]. As a result, the overall response rate to GEM monotherapy is less than 20%, and approximately 80% of patients succumb to the disease within one year due to recurrence [10]. The rapid development of resistance to GEM in PDAC patients is the primary limitation of GEM therapy. Most patients develop resistance to GEM within weeks of initiating its treatment, which significantly hampers the prognosis of PDAC patients [11]. Thus, understanding the mechanisms underlying GEM resistance is critical for the development of new drugs for the intervention of PDAC.

Several growth factor receptors, such as EGFR, VEGFR, PDGFR, and IGF-1R, are overexpressed in PDAC and promote tumor progression [12, 13]. A recent study further demonstrated that fibroblast growth factor receptors (FGFRs) represent the most upregulated receptor tyrosine kinases (RTKs) in certain gemcitabine-resistant PDAC cells, implicating their role in acquired gemcitabine resistance [14]. Additionally, clinical observations and our own data indicate that high levels of FGFR2 and FGFR3 correlate with advanced tumor stages and poor prognosis [15]. Therefore, we have focused on FGFR signaling as a potential therapeutic target in gemcitabine-resistant PDAC. However, FGFR activation alone does not fully account for the phenotypic changes observed during acquired gemcitabine resistance. Recent studies have highlighted the broader role of mucin-related pathways in PDAC chemotherapy resistance [16]. In particular, the MUC5AC is frequently upregulated in PDAC patients with poor prognosis and has been shown to shield cancer cells from cytotoxic agents [17]. Notably, MUC5AC expression is transcriptionally regulated by inflammatory pathways such as MAPK and NF-κB, which are canonical downstream effectors of FGFR signaling [18]. However, whether FGFR inhibition can modulate MUC5AC expression and thereby overcome gemcitabine resistance in PDAC remains unknown and constitutes a key focus of this study.

Derazantinib (ARQ-087) is a potent, orally bioavailable, ATP-competitive inhibitor of FGFRs 1 to 3 [19]. It has demonstrated significant in vitro and in vivo inhibitory effects on a variety of FGFR-dependent xenograft tumor models. Importantly, derazantinib has shown clinically meaningful efficacy and safety, with durable objective responses in clinical studies of intrahepatic cholangiocarcinoma [20].

In this study, we investigated whether treatment with derazantinib could alter malignant behaviors of GEM-resistant PDAC cells in vitro and tumors in vivo, as well as its action in inhibiting the FGFR-related MAPK and NF-κB signaling to regulate MUC5AC expression in GEM-resistant PDAC cells. We tested the hypothesis that FGFR may represent a promising therapeutic target for the intervention of GEM-resistant PDAC and that the combination of GEM and derazantinib may have the potential for clinical translation in the treatment of FGFR-related GEM-resistant patients.

## Materials and methods

### Establishment of gemcitabine resistant cell lines

Human PDAC AsPC-1 and BxPC-3 cell lines were from Cellcook (Guangzhou, China) and identified by STR. To establish stable GEM resistance, these cells were exposed to 50 nM GEM (MCE, Shanghai, China) for 24 h. Later, the media was discarded and the cells were fed with fresh 10% FBS-containing DMEM media to attain confluence. The GEM shock procedure was repeated 8–10 times with stepwise increase in GEM concentrations which resulted in the gradual increase of the drug concentration toleration threshold. After the cells grew stably at 50 nM, the GEM concentration was increased stepwise (50 → 100 → 200 → 300 → 500 nM). The induction lasted about 9 months, until the cells could grow stably in 500 nM GEM.

### Screening of the FDA-approved compound library

To screen potential drugs, AsPC-1 and BxPC-3 cells (5 × 10^3^ cells/well) were cultured in DMEM with 10% fetal bovine serum (complete medium) in a 96-well plate overnight. The cells were treated with 10 µM each of the FDA-approved 86 compounds in the library (Plate layout: PHD022932, TagetMol, Boston, MA, USA) for 48 h, as listed in Supplementary Table 1. Their cell viability was determined by CCK-8 assay using a CCK-8 kit and the inhibition rate of each candidate was calculated. The experiment was repeated three times.

### Patient specimens

Individual PDAC patients (*n* = 56) were recruited from our hospital. Inclusion criteria were (1) postoperative pathological diagnosis of PDAC; (2) availability of pathological tissue samples; and (3) no history of radiotherapy. The exclusion criteria encompassed ninety-day mortality, metastatic disease at the time of surgical resection, gross macroscopic positive resection margin (R2), and patients with incomplete follow-up data due to loss of follow-up within six months of surgery or incomplete medical records. According to the Response Evaluation Criteria in Solid Tumors, 18 patients receiving GEM-based treatment were categorized as GEM resistant (progressive disease) or GEM sensitive (partial response or Complete response in 1 year at least). This study was approved by the Ethics Committee of Zhejiang Cancer Hospital (Approval No.IRB-2023-470).

### Immunohistochemistry

The levels of FGFR2, FGFR3, and MUC5AC expression in individual PDAC tissue sections were examined by Immunohistochemistry (IHC) using specific antibodies (1:500 dilution) [14]. Their protein expression levels were quantified using the Histochemistry score (H-score), based on the proportion of positive cells in each section and their staining intensity. The H-Score was calculated as follows: H-Score (∑(pi×i)=(percentage of weak intensity cells×1) + (percentage of moderate intensity cells×2) + (percentage of strong intensity cells×3).

### Animal experiments

The animal study protocol was approved by the Ethics Committee of Zhejiang Cancer Hospital (Approval No.2024-09-02). female BALB/c nude mice at 4-week-old were from Charles River (zhejiang, china) and housed in a specific pathogen-free facility. Individual mice were implanted subcutaneously with 1 × 10^7^ BxPC-3(GR) cells. After tumor formation, the tumor-bearing mice were randomized and treated with vehicle saline (control), intraperitoneally with GEM (25 mg/kg body weight) and/or orally with derazantinib (50 mg/kg) every three days by gavage upon 21 days after the treatment beginning. The dynamic growth of tumors in individual mice was longitudinally monitored. Their tumor volumes were measured using a vernier caliper, and calculated as (length × width²)/2. At the end of the experiment, all mice were euthanized, and their subcutaneous tumors were dissected, measured, and fixed.

### Western blotting

The levels of protein expression in individual tumor samples and cells were quantified by Western blotting [21]. The specific antibodies included anti-FGFR1 (P11362); anti-FGFR2 (P11362) and anti-FGFR3 (P22607, Baijia, Taizhou, China), anti-FGFR2 (YT0485) and anti-NF-κB P105/P50 (PT0463R, Immunoway, Suzhou, China) anti-MUC5AC (30408-1-AP) and anti-ERK1/2 (66192-1-Ig, Proteintech, Wuhan, China); anti-SAPK/JNK (#9252) and anti-phospho-SAPK/JNK (#9251, Cell Signaling Technology, Shanghai, China); anti-IKBα (#ET1603-6) and anti-NF-κB P65 (#ET1603-12, HUABIO, Hangzhou, China). These primary and secondary antibodies were used at 1:2000 and 1:5000, respectively.

### Statistical analysis

Data are presented as mean ± standard deviation (SD). The difference between groups was analyzed using Student’s t-test. The relationship between two groups was determined by Spearman’s correlation coefficient. The signaling pathway enrichment was analyzed using Metascape. The distribution of different variants was generated by heatmaps using TBtools-II v2.210 and their relationship was analyzed by Venn diagrams using EVenn. The enrichment of potential signaling pathways was analyzed by volcano plots, KEGG pathway analysis, and TRRUST enrichment analyses using GraphPad Prism 9.5 (Win11) and Compusyn software (Win11).

Expression profile data were obtained from GEO database (GSE140077, GSE152121, and GSE79953). The association of MUC5AC mRNA with prognosis of PDAC was analyzed by Kaplan-Meier Plotter, GEPIA2, and GEO database. The correlation of FGFR2, MUC5AC, RELA, and ERK1 mRNA transcripts in PDAC tissues from GEPIA2 and cBioPortal databases was analyzed. A *p*-value of < 0.05 was considered statistically significant.The protein-protein interaction network of the differentially expressed genes (DEGs) was analyzed by STRING database and Cytoscape v3.10.3.

## Results

### Derazantinib treatment inhibits the growth and reduces the IC50 of GEM in GEM-resistant PDAC cells by attenuating FGFR2 and FGFR3 expression

**Fig. 1:**
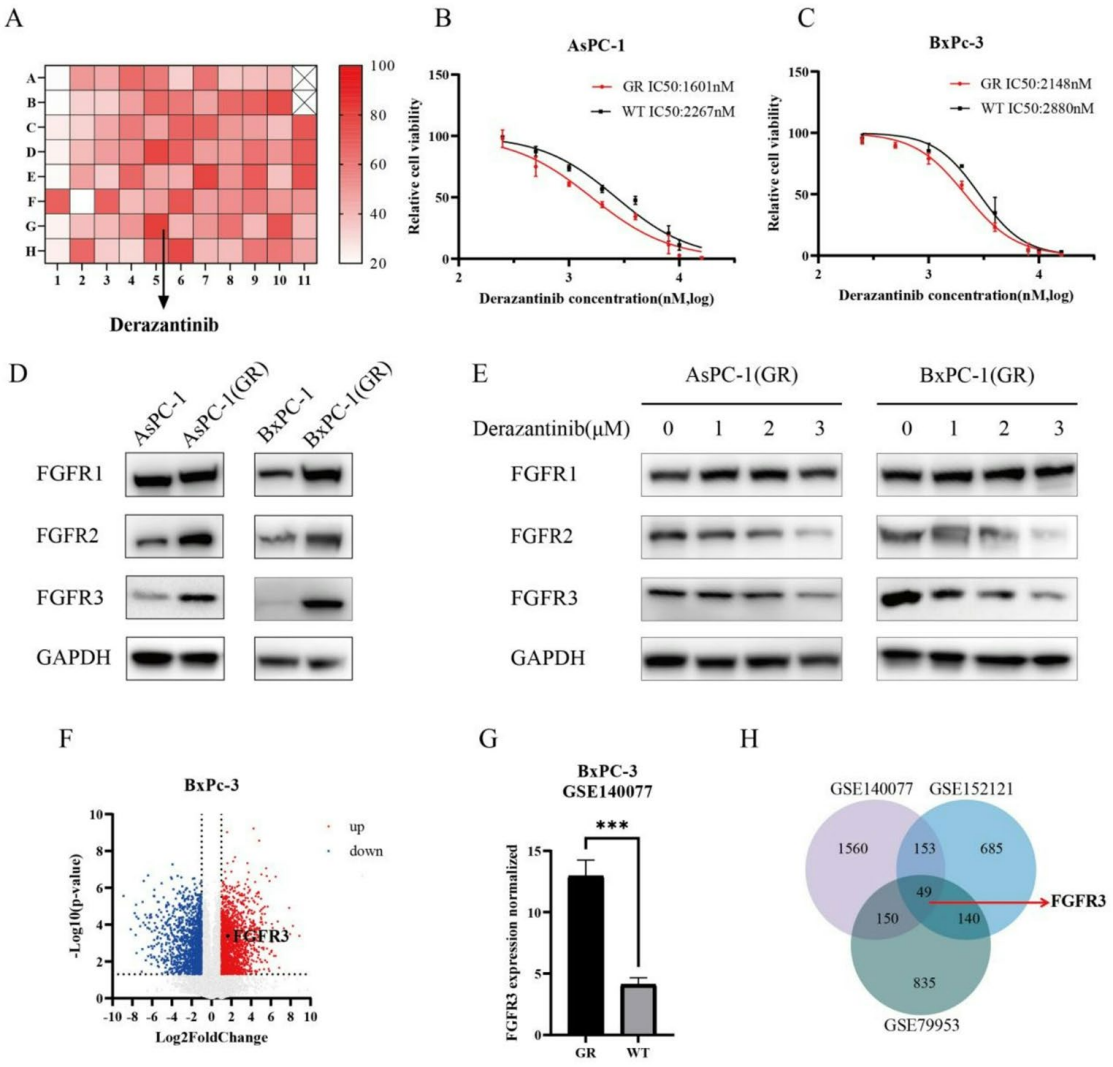
Derazantinib is a potential agent against gemcitabine-resistant PDAC. (**A**) Inhibitory effects of compounds at an equal concentration from the drug library on PDAC cells. (**B**) The IC50 values of derazantinib in AsPC-1(GR) and AsPC-1(WT) cells. (**C**) The IC50 values of deazantinib in BxPC-3(GR) and BxPC-3(WT) cells. (**D**) Western blot analysis of the levels of FGFR1-3 protein expression in wild-type and GEM-resistant cells. (**E**) Western blot analysis of the levels of FGFR1-3 protein expression in AsPC-1(GR) and BxPC-3(GR) cells following treatment with different concentrations of derazantinib. (**F**) Volcano plot presence of differentially expressed genes between BxPC-3(GR) and derazantinib-treated BxPC-3(GR) cells in GEO dataset. (**G**) FGFR3 mRNA transcripts in BxPC-3(WT) and BxPC-3(GR) cells from GEO dataset. (**H**) The Venn diagram of GEM-resistant genes in PDAC was analyzed by intersecting GEO datasets (GSE140077、GSE152121 and GSE79953). The data are shown as mean ± SD. *p < 0.05; **p < 0.01; ***p < 0.001 by Student’s t test

To identify the potential candidates for the treatment of PDAC, PDAC cells were treated with individual candidate drugs for 48 h (Table S1) and their inhibitory rates were measured by CCK-8 assays. The results indicated that derazantinib exhibited the highest average inhibition rate among all candidate drugs (Figure 1 A). Next, we generated GEM-resistant AsPC-1 and BxPC-3 cells (AsPC-1(GR) and BxPC-3(GR)) (Figures S1A and S1B). Derazantinib remained the most effective candidate in the drug library, and the resistance to derazantinib was slightly lower in GEM-resistant cells than in wild-type cells assessed by IC50 values (Figure 1 C, S1B and S1C). Compared with their parental counterparts, the protein levels of FGFR1, FGFR2 and FGFR3 were elevated in the AsPC-1(GR) and BxPC-3(GR) cells (Fig. 1D). Upon treatment with derazantinib, the expression of FGFR2 and FGFR3—but not FGFR1—was reduced in a dose-dependent manner in both resistant cell lines (Fig. 1E). Further analysis of differentially expressed genes between BxPC-3 and BxPC-3(GR) unveiled FGFR3 mRNA transcripts were significantly elevated in BxPC-3(GR) cells, relative to that in BxPC-3 cells (Fig. 1F and H). The up-regulated FGFR3 expression contributed to the development of GEM resistance and targeting FGFR3 and FGFR2 by derazantinib effectively reduced the resistance to GEM in PDAC cells.

### Higher levels of FGFR expression are associated with worse survival of PDAC patients

**Fig. 2:**
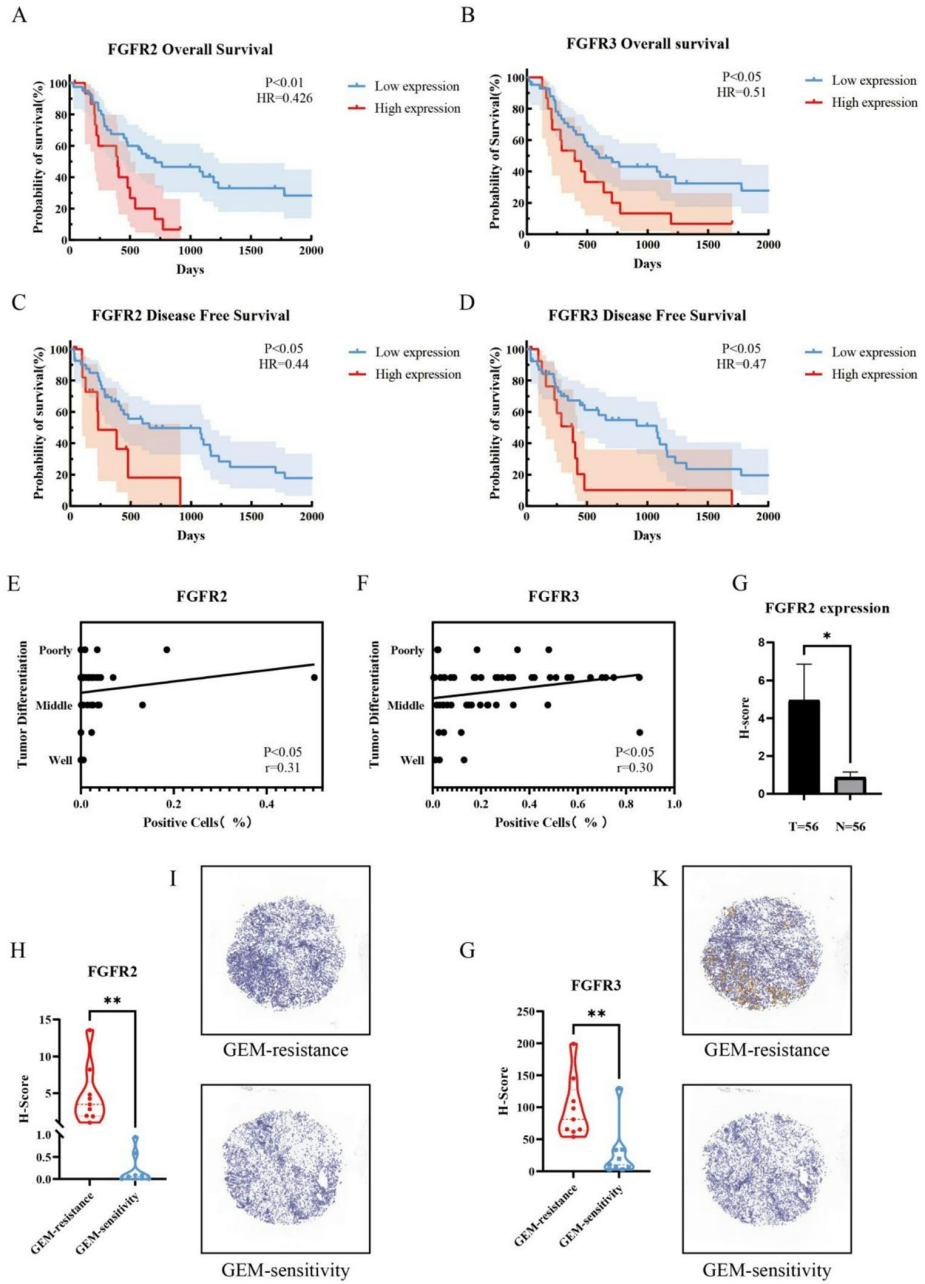
Negative association between FGFR expression and clinical outcomes in PDAC patients. (**A**,** B**) High levels of FGFR2 and FGFR3 expression in PDAC tissues by IHC staining were negatively associated with worse overall survival (OS) of patients. (**C**,** D**) High levels of FGFR2 and FGFR3 expression in PDAC tissues by IHC staining were negatively associated with worse disease-free survival (DFS) of patients. (**E**,** F**) The percentages of FGFR2 + and FGFR3 + cells by IHC staining were negatively correlated with the degree of PDAC differentiation. (**G**) FGFR2 expression was upregulated in PDAC, compared with the adjacent tissue. (**H**) FGFR2 was highly expressed in GEM-resistant PDAC tissues. (**I**) Representative images of IHC staining for FGFR2 expression in GEM-resistant and GEM-sensitive PDAC tissues. (**J**) FGFR3 was highly expressed in GEM-resistant PDAC tissues. (**K**) Representative images of IHC staining for FGFR3 expression in GEM-resistant and GEM-sensitive PDAC tissues. The data are shown as mean ± SD. *p < 0.05; **p < 0.01; ***p < 0.001 by Student’s t test

To investigate the clinical significance of FGFR expression in PDAC, we analyzed FGFR2 and FGFR3 expression in 56 PDAC tissues of patients at our center by immunohistochemistry (IHC). The results indicated that the higher levels of FGFR2 and FGFR3 expression were negatively associated with shorter overall survival (OS) and disease-free survival of those patients (*p* < 0.05 or *p* < 0.01, Fig. 2A and D). Furthermore, the percentages of positive FGFR2 and FGFR3 tumor cells were negatively associated with the degrees of tumor differentiation (Fig. 2E and F). Compared with adjacent non-tumor tissues, FGFR2 expression was significantly up-regulated in 56 PDAC tissues (Fig. 2G). Analysis of FGFR expression levels in nine pairs of GEM-resistant and GEM-sensitive tissue samples from our center revealed that FGFR2 and FGFR3 expression in GEM-resistant PDAC tissues. (Figure 2H and K). These data further indicated that FGFR2 and FGFR3 were therapeutic targets for the intervention of GEM-resistant PDAC tissues.

### Treatment with derazantinib and GEM synergistically inhibits the malignant behaviors of GEM-resistant PDAC cells in vitro

**Fig. 3:**
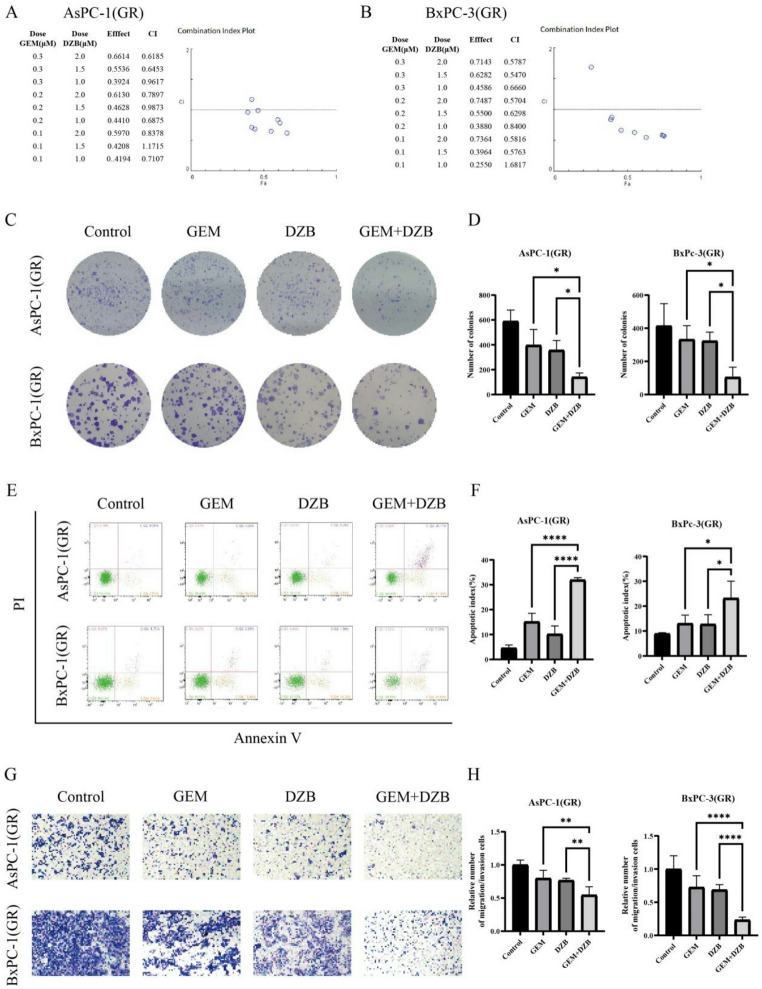
Synergistic antitumor activity of derazantinib with gemcitabine in gemcitabine-resistant PDAC cells. (**A**,** B**) The combination index of GEM and derazantinib in AsPC-1(GR) and BxPC-3(GR) cells. (**C**,** D**) Treatment with GEM and derazantinib synergistically reduced the number of colonies in GEM-resistant cells. (**E**,** F**) Treatment with GEM and derazantinib increased the percentages of apoptotic GEM-resistant cells. (**G**,** H**) Treatment with GEM and derazantinib further inhibited the migration of GEM-resistant cells. The data are shown as mean ± SD. *p < 0.05; **p < 0.01; ***p < 0.001 by Student’s t test

To evaluate the potential effect of combining derazantinib with GEM, we assessed the combination index (CI) of the two drugs in GEM-resistant PDAC cells. Our results revealed a synergistic therapeutic effect of derazantinib and GEM (Fig. 3A and B). Furthermore, treatment with both derazantinib and GEM synergistically reduced the number of tumor cell colonies in both AsPC-1(GR) and BxPC-3(GR) cells while such treatment dramatically increased the percentages of apoptotic AsPC-1(GR) and BxPC-3(GR) cells, relative to that of cells treated with single drug alone (Fig. 3C-F). In addition, transwell assays displayed that treatment with both derazantinib and GEM significantly decreased the number of migrated AsPC-1(GR) and BxPC-3(GR) cells (Fig. 3G and H). Collectively, these data clearly indicated that treatment with derazantinib and GEM synergistically inhibited the malignant behaviors of GEM-resistant PDAC cells in vitro.

### MUC5AC played a significant role in the DEGs and was linked to poor prognosis and chemotherapy resistance

**Fig. 4:**
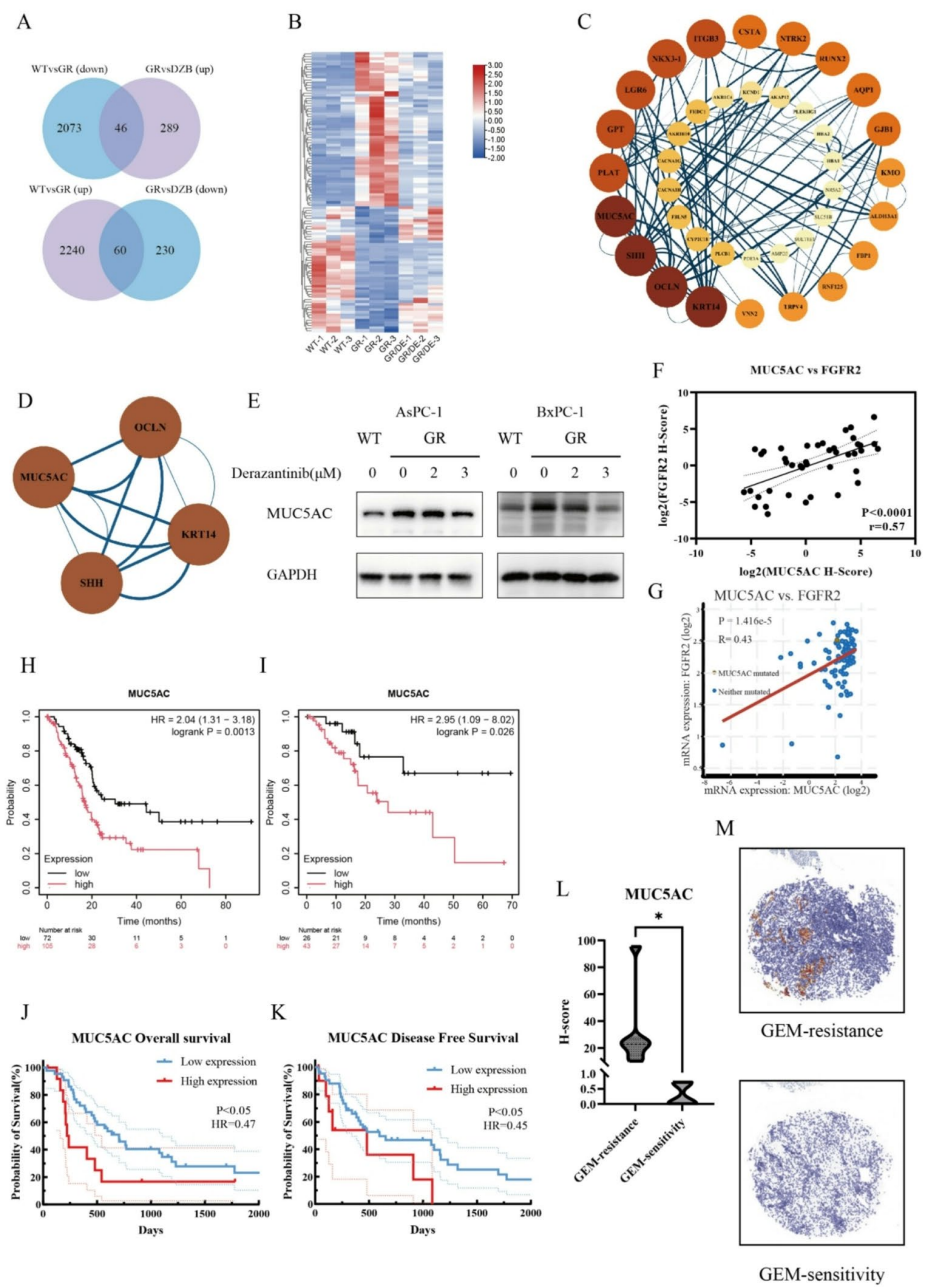
MUC5AC and FGFR are key factors in PDAC progression and chemotherapy resistance are linked to poor prognosis. (**A**) Venn diagram depicts the number of the DEGs in the WT/GR and GR/DZB groups. (**B**) Heatmap analysis of DEGs. (**C**) Protein-protein interaction networks of the key DEGs. (**D**) Screening of key DEGs. (**E**) Western blot analysis of the levels of MUC5AC protein expression in wild-type and GEM-resistant cells following derazantinib treatment with the indicated concentration. (**F**) The levels of MUC5AC were positively correlated with FGFR2, analyzed by IHC. (**G**) The levels of MUC5AC were positively correlated with FGFR2, analyzed by cBioPortal. (**H-I**) Kaplan-Meier Plotter analysis revealed that the levels of MUC5AC expression were negatively associated with the survival of PDAC patients. (**J-K**) The levels of MUC5AC protein expression by IHC were negatively associated with the survival of PDAC patients. (**L**) MUC5AC was highly expressed in GEM-resistant tissues. (**M**) Representative images of IHC staining for MUC5AC expression in GEM-resistant and GEM-sensitive PDAC tissues. The data are shown as mean ± SD. *p < 0.05; **p < 0.01; ***p < 0.001 by Student’s t test

To investigate the mechanisms underlying the action of derazantinib in PDAC cells, the transcription profiles of WT, AsPC-1(GR), and AsPC-1(GR) cells had been treated with derazantinib and were analyzed by RNA-seq. Compared with the WT AsPC-1 cells, 60 DEGs were upregulated and 46 DEGs were down-regulated in AsPC-1(GR) (Fig. 4A and B). In contrast, the up-regulated 60 DEGs in AsPC-1(GR) cells were significantly mitigated or abrogated in the derazantinib-treated while the and 46 down-regulated DEGs in AsPC-1(GR) cells were restored in the derazantinib-treated AsPC-1(GR) cells. The protein-protein interaction (PPI) network analysis of the DEGs using the STRING database and Cytoscape revealed that MUC5AC was a key node induced by derazantinib in GEM-resistant PDAC cells (Fig. 4C and D). Western blot analysis exhibited that the levels of MUC5AC protein expression were upregulated in AsPC-1(GR) and BxPC-3(GR) cells but decreased by treatment with increased concentrations of derazantinib (Fig. 4E). To further evaluate the role of MUC5AC in PDAC, we analyzed its impact on patient prognosis and its association with FGFR expression in human PDAC tissues. The data indicated that levels of MUC5AC expression were positively correlated with the levels of FGFR IHC H-score or mRNA transcripts in PDAC tissues (Fig. 4F and G) and higher levels of MUC5AC expression in PDAC tissues were associated with worse OS and DFS in PDAC patients (Fig. 4H and K). Finally, the levels of MUC5AC expression in GEM-resistant PDAC tissues were significantly higher than those in the GEM-sensitive PDAC tissues from our center (Fig. 4L and M). Hence, derazantinib treatment attenuated the up-regulated MUC5AC expression in GEM-resistant PDAC tumors.

### Derazantinib inhibits MUC5AC expression by attenuating the NF-κB and MAPK signaling in PDAC cells

**Fig. 5:**
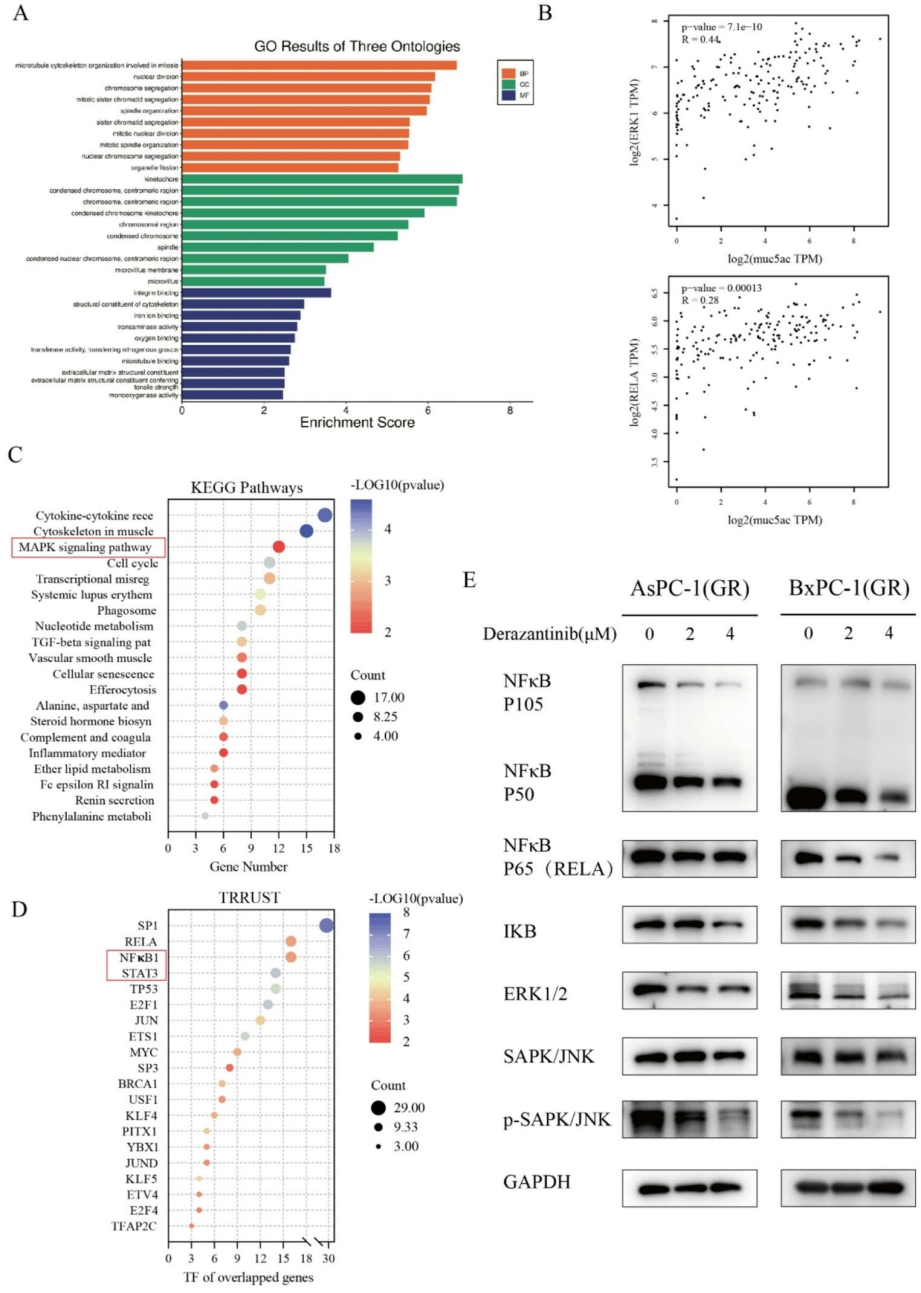
Derazantinib inhibits MUC5AC via NF-κB and MAPK signaling pathways. (**A**) GO cellular component enrichment analyses of DEGs from GR/DZB groups. (**B**) Correlation analysis between gene expression levels (ERK1, MUC5AC, RELA). (**C**) The KEGG pathway enrichment analysis of DEGs from GR/DZB groups. (**D**) The TRRUST enrichment analysis of DEGs from GR/DZB groups. (**E**) Derazantinib treatment reduced the levels of NF-κB P105/P50, NF-κB P65, IKB, ERK1/2, SAPK/JNK and p-SAPK/JNK protein expression in the AsPC-1(GR) and BxPC-3(GR) cells

To understand how derazantinib inhibited the malignant behaviors of GEM-resistant PDAC cells, we further analyzed the DEGs (GR vs. DZB). GO enrichment analysis indicated that the enriched DEGs were involved in various biological processes, including regulation of cell cycle progression and cell cycle phase transition (Fig. 5A), consistent with the synergistic effect of derazantinib and GEM on inhibiting the proliferation of PDAC cells.

Previous studies have shown that MUC5AC expression is positively regulated by the MAPK and NF-κB signaling [18, 22–24]. Actually, the GEPIA2 dataset analysis exhibited that MUC5AC mRNA transcripts were positively correlated with the levels of ERK1 and RELA mRNA transcripts in PDAC tumors (Fig. 5B). Subsequent KEGG pathway enrichment analysis and TRRUST regulatory network analysis further supported that DEGs were enriched in the MAPK and NF-κB pathways (Fig. 5C and D).

To validate these findings, we evaluated the expression levels of key markers in the MAPK and NF-κB pathways by Western blotting. We found that treatment with derazantinib notably reduced the levels of ERK1/2, p-SAPK/JNK, NF-κB P105/P50, NF-κB P65, and IKBα expression and SAPK/JNK phosphorylation in AsPC-1(GR) and BxPC-3(GR) cells (Fig. 5E). These results support the hypothesis that derazantinib may inhibit MUC5AC expression by attenuating the FGFR/MAPK and FGFR/NF-κB signaling to suppress the malignant behaviors of GEM-resistant PDAC cells.

### Treatment with derazantinib and GEM synergistically inhibits the growth of GEM-resistant BxPC-3(GR) tumors in mice

**Fig. 6:**
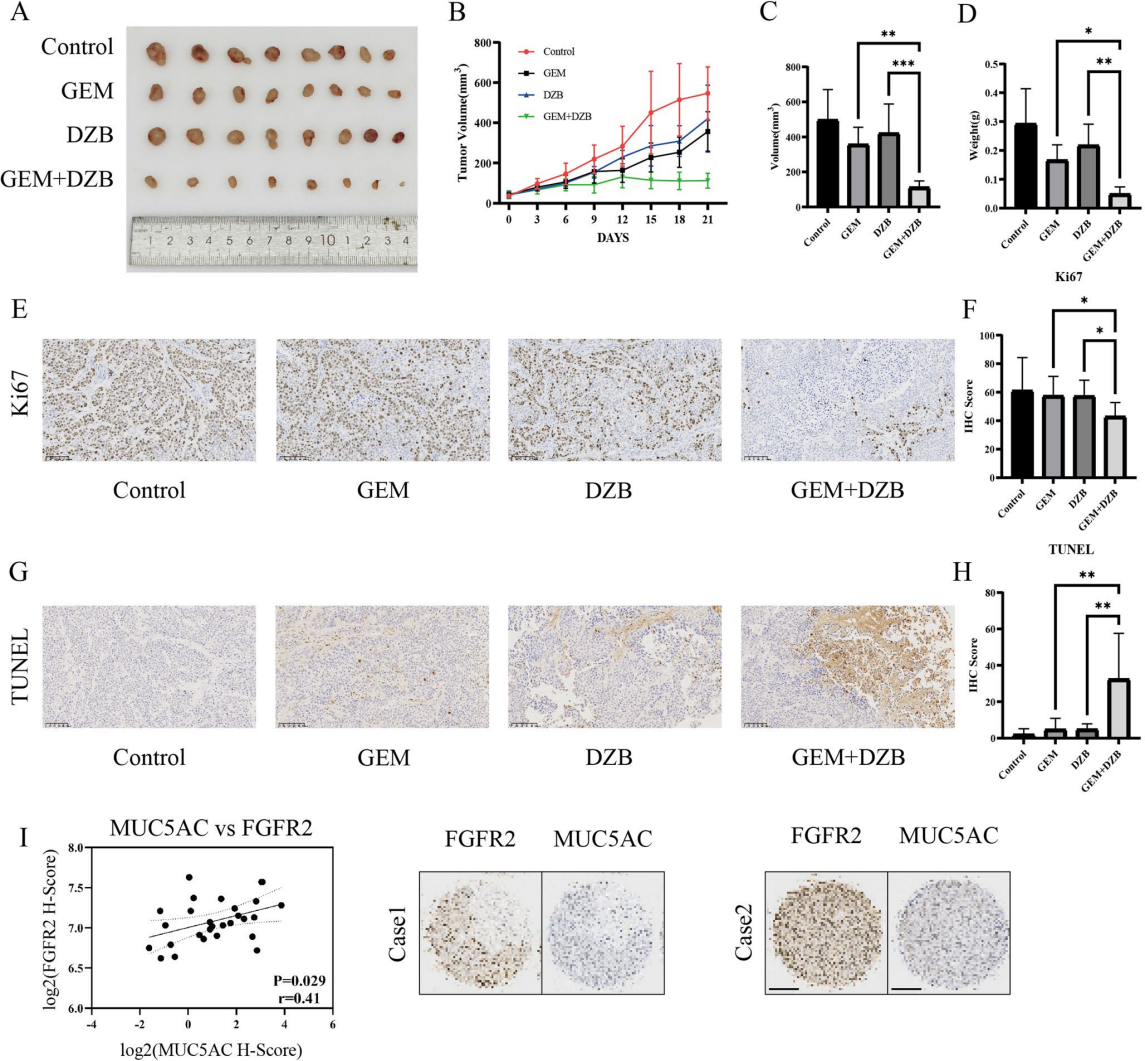
Derazantinib sensitizes BxPC-3/GR cells-derived xenografts to GEM treatment. (**A**) Tumor images from Control, GEM, DZB and GEM + DZB groups. (**B**) Tumor growth curves of each group. (**C**) Tumor volumes of each group. (**D**) Tumor weights of each group. (**E**,** F**) Representative IHC staining of Ki67 expression in tumors from different groups. (**G**,** H**) Representative IHC staining of TUNEL + cells in tumors from different groups. (**I**) Representative images and protein H-score correlation plot of MUC5AC and FGFR2 in tumor tissues. The data are shown as mean ± SD. *p < 0.05; **p < 0.01; ***p < 0.001 by Student’s t test

Finally, we investigated whether treatment with derazantinib could enhance the sensitivity of GEM-resistant PDAC to GEM in vivo. BALB/c nude mice were implanted subcutaneously with BxPC-3(GR) cells to induce solid tumors. After the establishment of solid tumors, the mice were randomized and treated with vehicle, GEM and/or derazantinib every three days for 3 weeks. Their tumor growth was monitored longitudinally (Fig. 6A) and their tumors were dissected, followed by photoimaging (Fig. 6B). Compared with the vehicle control group, treatment with GEM or derazantinib slightly inhibited the growth of implanted BxPC-3(GR) tumors and reduced their sizes and treatment with both GEM and derazantinib further inhibited the growth of BxPC-3(GR) tumors and decreased their sizes. As a result, treatment with both GEM and derazantinib significantly decreased tumor volumes and weights, relative to that in the mice with a monotherapy (Fig. 6C and D). IHC staining revealed that treatment with both GEM and derazantinib dramatically decreased the percentages of KI67 + cells and increased the percentages of apoptotic cells in tumor tissues (Figs. 6E-H). Additionally, the MUC5AC and FGFR2 protein expression levels were positively correlated in the tumor tissues (Figures 6I). Together, these data implied that treatment with derazantinib significantly enhanced the sensitivity of GEM-resistant PDAC tumors to GEM chemotherapy.

## Discussion

Currently, GEM is the baseline chemotherapeutic drug for PDAC [25, 26]. However, resistance to GEM remains one of the most significant challenges in treating PDAC [27]. Overcoming drug resistance has been a major goal of numerous studies aimed at elucidating the molecular mechanisms of drug resistance and developing therapeutic strategies for PDAC.

A previous study has shown that FGFR is highly expressed in drug-resistant PDAC cells and patients [28]. FGFR is an upstream receptor tyrosine kinase (RTK) that contributes to drug resistance and has been generally considered a druggable target [29, 30]. Several pan-FGFR inhibitors have been demonstrated to have single-agent efficacy in a variety of cancers, including PDAC [31–34], particularly in patients with FGFR mutations [35]. Derazantinib is primarily characterized as an ATP-competitive inhibitor targeting FGFR1-4, suppressing ligand-dependent receptor phosphorylation [19]. In our study, we additionally observed a reduction in total FGFR2 and FGFR3 protein levels following derazantinib treatment in PDAC cells. Notably, accumulating evidence indicates that certain RTK-targeted inhibitors can modulate receptor abundance beyond kinase blockade [36, 37]. These agents may facilitate receptor ubiquitination, internalization, and subsequent lysosomal or proteasomal degradation, ultimately leading to decreased total protein levels [38, 39]. For example, a recent study demonstrated that erdafitinib induces TRIM25-dependent ubiquitination of FGFR1/4, leading to receptor internalization and degradation [40]. Given that derazantinib belongs to the same class of FGFR-directed small-molecule inhibitors, a similar mechanism may underlie the downregulation of FGFR2/3 observed in our models.

Inhibition of FGFR1 has been reported to enhance the efficacy of chemotherapy for PDAC [14]. Given the association of FGFR2 and FGFR3 with poor prognosis and GEM resistance in our PDAC cohort, we investigated the synergistic effects of derazantinib with GEM in both in vitro and in vivo GEM-resistant PDAC models. The results indicated that derazantinib treatment overcame GEM resistance in PDAC cells and synergistically with GEM inhibited the malignant behaviors of PDAC cells in vitro and PDAC tumors in vivo.

Previous studies have shown that MUC5AC is associated with poor prognosis and drug resistance in PDAC and can be activated through the MAPK and NF-κB pathways [17, 41]. In this study, we found that derazantinib treatment reduced the expression of MUC5AC in GEM-resistant PDAC cells. Mechanistically, derazantinib treatment significantly attenuated the levels of MAPK and NF-κB signaling event expression and SAPK/JNK phosphorylation in GEM-resistant PDAC cells. It is possible that derazantinib treatment inhibited the FGFR-related MAPK and NF-κB signaling to suppress MUC5AC expression, contributing to restoring GEM sensitivity in PDAC cells. We are interested in further investigating how derazantinib treatment overcomes GEM-resistance in PDAC.

FGFR is crucial for the development of drug resistance in PDAC. Our data highlighted the role of FGFR-related GEM resistance in PDAC. FGFRi may also have prognostic potential to identify existing or developing GEM resistance. GEM is widely used in numerous malignancies, including cholangiocarcinoma, breast, ovarian, non-small cell lung and bladder cancers [42, 43]. Therefore, derazantinib to inhibit the FGFR-related signlaing may enhance the efficacy of GEM in additional types of cancers.

## Conclusion

Our data indicated that the FGFR2 and FGFR3 are crucial for the progression and drug resistance of PDAC. Evidently, derazantinib treatment effectively inhibited the malignant behaviors of drug-resistant PDAC cells and enhanced the sensitivity to GEM through attenuating the NF-κB and MAPK pathways to inhibit MUC5AC expression. These findings suggest that derazantinib may be a promising chemotherapeutic adjuvant for treating PDAC, particularly for patients with GEM-resistant PDAC. The findings may provide a basis for translating derazantinib into clinical trials to improve the efficacy of GEM in PDAC patients. Conceivably, targeting FGFR and MUC5AC may be a novel strategy to overcome GEM resistance and to improve therapeutic efficacy in PDAC patients.

## Supplementary Information

Below is the link to the electronic supplementary material.

Supplementary Material 1

## Data Availability

The datasets used and/or analyzed in the current study are available from the corresponding author upon a reasonable request.

## References

[1] Stoffel EM, Brand RE, Goggins M. Pancreatic cancer: changing epidemiology and new approaches to risk assessment, early detection, and prevention. Gastroenterology. 2023;164(5):752–65. 36804602 10.1053/j.gastro.2023.02.012 PMC10243302

[2] Zhou Y, et al. Burden of six major types of digestive system cancers globally and in China. Chin Med J (Engl). 2024;137(16):1957–64. 38958046 10.1097/CM9.0000000000003225 PMC11332782

[3] Siegel RL, et al. Cancer statistics, 2025. CA Cancer J Clin. 2023;75(1):10–45. 10.3322/caac.21871 PMC11745215 39817679

[4] Gyawali B, Booth CM. Treatment of metastatic pancreatic cancer: 25 years of innovation with little progress for patients. Lancet Oncol. 2024;25(2):167–70. 38301687 10.1016/S1470-2045(23)00516-8

[5] Gemenetzis G, et al. Survival in locally advanced pancreatic cancer after neoadjuvant therapy and surgical resection. Ann Surg. 2019;270(2):340–7. 29596120 10.1097/SLA.0000000000002753 PMC6985003

[6] Mizrahi JD, et al. Pancreatic cancer. Lancet. 2020;395(10242):2008–20. 32593337 10.1016/S0140-6736(20)30974-0

[7] Koltai T, et al. Resistance to gemcitabine in pancreatic ductal adenocarcinoma: a physiopathologic and pharmacologic review. Cancers (Basel). 2022. 10.3390/cancers14102486. 35626089 10.3390/cancers14102486 PMC9139729

[8] Beutel AK, Halbrook CJ. Barriers and opportunities for gemcitabine in pancreatic cancer therapy. Am J Physiol Cell Physiol. 2023;324(2):C540–52. 36571444 10.1152/ajpcell.00331.2022 PMC9925166

[9] Jiang X, et al. Targeting UBE2T potentiates gemcitabine efficacy in pancreatic cancer by regulating pyrimidine metabolism and replication stress. Gastroenterology. 2023;164(7):1232–47. 36842710 10.1053/j.gastro.2023.02.025

[10] Yang G, et al. Integrative genomic analysis of gemcitabine resistance in pancreatic cancer by Patient-derived xenograft models. Clin Cancer Res. 2021;27(12):3383–96. 33674273 10.1158/1078-0432.CCR-19-3975

[11] Binenbaum Y, Na’ara S, Gil Z. Gemcitabine resistance in pancreatic ductal adenocarcinoma. Drug Resist Updat. 2015;23:55–68. 26690340 10.1016/j.drup.2015.10.002

[12] Valsecchi ME, et al. Epidermal growth factor receptor and insulinlike growth factor 1 receptor expression predict poor survival in pancreatic ductal adenocarcinoma. Cancer. 2012;118(14):3484–93. 10.1002/cncr.26661. 22086503 10.1002/cncr.26661

[13] Angelescu R, et al. Expression of vascular endothelial growth factor and epidermal growth factor receptor in pancreatic ductal adenocarcinomas, neuroendocrine tumours and chronic pancreatitis. Endosc Ultrasound. 2013;2(2):86–91. 24949370 10.4103/2303-9027.117692 PMC4062250

[14] Lin Q, et al. Fibroblast growth factor receptor 1 Inhibition suppresses pancreatic cancer chemoresistance and chemotherapy-driven aggressiveness. Drug Resist Updat. 2024;73:101064. 38387284 10.1016/j.drup.2024.101064 PMC11864563

[15] Kang X, et al. Deciphering role of FGFR signalling pathway in pancreatic cancer. Cell Prolif. 2019;52(3):e12605. 30945363 10.1111/cpr.12605 PMC6536421

[16] Dieli R, et al. The oncoprotein mucin 1 in pancreatic cancer onset and progression: potential clinical implications. Biomolecules. 2025;15(2):275. 40001578 10.3390/biom15020275 PMC11853026

[17] Ganguly K, et al. Mucin 5AC serves as the nexus for beta-Catenin/c-Myc interplay to promote glutamine dependency during pancreatic cancer chemoresistance. Gastroenterology. 2022;162(1):253–68. e13. 34534538 10.1053/j.gastro.2021.09.017 PMC8678212

[18] Liou CJ, Huang WC. Casticin inhibits interleukin-1beta-induced ICAM-1 and MUC5AC expression by blocking NF-kappaB, PI3K-Akt, and MAPK signaling in human lung epithelial cells. Oncotarget. 2017;8(60):101175–88. 29254155 10.18632/oncotarget.20933 PMC5731865

[19] Raggi C. Antitumor activity of a novel fibroblast growth factor receptor inhibitor for intrahepatic cholangiocarcinoma. Am J Pathol. 2019;189(10):2090–101. 31351075 10.1016/j.ajpath.2019.06.007

[20] Braun S, et al. Derazantinib: an investigational drug for the treatment of cholangiocarcinoma. Expert Opin Investig Drugs. 2021;30(11):1071–80. 34698609 10.1080/13543784.2021.1995355

[21] Li X, et al. Thiol oxidative stress-dependent degradation of transglutaminase2 via protein S-glutathionylation sensitizes 5-fluorouracil therapy in 5-fluorouracil-resistant colorectal cancer cells. Drug Resist Updat. 2023;67:100930. 36736043 10.1016/j.drup.2023.100930

[22] Lee SU, et al. Transforming growth factor beta inhibits MUC5AC expression by Smad3/HDAC2 complex formation and NF-kappaB deacetylation at K310 in NCI-H292 cells. Mol Cells. 2021;44(1):38–49. 33510050 10.14348/molcells.2020.0188 PMC7854180

[23] Chen X, et al. Muc5ac production inhibited by decreased LncRNA H19 via PI3K/Akt/NF-κB in asthma. J Asthma Allergy. 2021;14:1033–43. 34421304 10.2147/JAA.S316250 PMC8373259

[24] Shin IS, et al. Melatonin inhibits MUC5AC production via suppression of MAPK signaling in human airway epithelial cells. J Pineal Res. 2014;56(4):398–407. 24720799 10.1111/jpi.12127

[25] Park W, Chawla A, O’Reilly EM. Pancreatic Cancer: Rev JAMA. 2021;326(9):851–62. 10.1001/jama.2021.13027 PMC9363152 34547082

[26] Conroy T, et al. Five-year outcomes of FOLFIRINOX vs gemcitabine as adjuvant therapy for pancreatic cancer: a randomized clinical trial. JAMA Oncol. 2022;8(11):1571–8. 36048453 10.1001/jamaoncol.2022.3829 PMC9437831

[27] Konate MM, et al. Insights into gemcitabine resistance in pancreatic cancer: association with metabolic reprogramming and TP53 pathogenicity in patient derived xenografts. J Transl Med. 2024;22(1):733. 39103840 10.1186/s12967-024-05528-6 PMC11301937

[28] Rasam S, et al. Highly reproducible quantitative proteomics analysis of pancreatic cancer cells reveals proteome-level effects of a novel combination drug therapy that induces cancer cell death via metabolic remodeling and activation of the extrinsic apoptosis pathway. J Proteome Res. 2023;22(12):3780–92. 37906173 10.1021/acs.jproteome.3c00463

[29] Ito Y, et al. The combination of gemcitabine plus an anti-FGFR inhibitor can have a synergistic antitumor effect on FGF-activating cholangiocarcinoma. Cancer Lett. 2024;595:216997. 38801887 10.1016/j.canlet.2024.216997

[30] Kawahara I, et al. Targeting metabolic reprogramming to overcome drug resistance in advanced bladder cancer: insights from gemcitabine- and cisplatin-resistant models. Mol Oncol. 2024;18(9):2196–211. 38874588 10.1002/1878-0261.13684 PMC11467791

[31] Papadopoulos KP, et al. A phase 1 study of ARQ 087, an oral pan-FGFR inhibitor in patients with advanced solid tumours. Br J Cancer. 2017;117(11):1592–9. 28972963 10.1038/bjc.2017.330 PMC5729432

[32] Abou-Alfa GK, et al. Pemigatinib for previously treated, locally advanced or metastatic cholangiocarcinoma: a multicentre, open-label, phase 2 study. Lancet Oncol. 2020;21(5):671–84. 32203698 10.1016/S1470-2045(20)30109-1 PMC8461541

[33] Loriot Y, et al. Erdafitinib or chemotherapy in advanced or metastatic urothelial carcinoma. N Engl J Med. 2023;389(21):1961–71. 37870920 10.1056/NEJMoa2308849

[34] Wainberg ZA, et al. Bemarituzumab in patients with FGFR2b-selected gastric or gastro-oesophageal junction adenocarcinoma (FIGHT): a randomised, double-blind, placebo-controlled, phase 2 study. Lancet Oncol. 2022;23(11):1430–40. 36244398 10.1016/S1470-2045(22)00603-9

[35] Ng CF, et al. Exceptional response to erdafitinib in FGFR2-Mutated metastatic pancreatic ductal adenocarcinoma. J Natl Compr Canc Netw. 2022;20(10):1076–9. 36240849 10.6004/jnccn.2022.7039

[36] Santorelli S, et al. In vivo effects of AZD4547, a novel fibroblast growth factor receptor inhibitor, in a mouse model of endometriosis. Pharmacology Research & Perspectives. 2021;9(2):e00759. 33811484 10.1002/prp2.759 PMC8019068

[37] Du G, Jiang J, et al. Discovery of a potent degrader for fibroblast growth factor receptor 1/2. Angew Chem Int Ed Engl. 2021;60(29):15905–11. 33915015 10.1002/anie.202101328 PMC8324087

[38] Zhu X, Wang L, et al. Erdafitinib promotes ferroptosis in human uveal melanoma by inducing ferritinophagy and lysosome biogenesis via modulating the FGFR1/mTORC1/TFEB signaling axis. Free Radic Biol Med. 2024;222:552–68. 38971541 10.1016/j.freeradbiomed.2024.07.002

[39] Szybowska P, et al. Negative regulation of FGFR (Fibroblast growth factor receptor) signaling. Cells. 2021;10(6):1342. 34071546 10.3390/cells10061342 PMC8226934

[40] Luo Q, et al. Erdafitinib inhibits the tumorigenicity of MDA-MB-231 triple-negative breast cancer cells by inducing TRIM25/ubiquitin-dependent degradation of FGFR4. Breast Cancer Res. 2025;27(1):128. 40635078 10.1186/s13058-025-02086-7 PMC12239495

[41] He Y, et al. Clinical efficacy and chemoresistance analysis of precision neoadjuvant chemotherapy for borderline resectable pancreatic cancer: a prospective, single-arm pilot study. Int J Surg. 2025. 10.1097/JS9.0000000000002342. 40146255 10.1097/JS9.0000000000002342 PMC12165568

[42] Rubnitz Z, et al. Gemcitabine-induced myositis in a patient with pancreatic cancer. BMJ Case Rep. 2025. 10.1136/bcr-2024-259660. 39842889 10.1136/bcr-2024-259660 PMC11759102

[43] Li K, et al. Neoadjuvant gemcitabine-cisplatin plus Tislelizumab in persons with resectable muscle-invasive bladder cancer: a multicenter, single-arm, phase 2 trial. Nat Cancer. 2024;5(10):1465–78. 39256488 10.1038/s43018-024-00822-0

