# Supplementary material for: Derazantinib enhances gemcitabine efficacy in PDAC by attenuating the NF-κB and MAPK pathways to suppress MUC5AC expression
Source: Med Oncol. 2025 Dec 30;43(2):107. doi: 10.1007/s12032-025-03222-1 (PMC12753570; doi:10.1007/s12032-025-03222-1)
Supplement: Supplementary file 1 — Supplementary Material 1 [file 12032_2025_3222_MOESM1_ESM.pdf]

Figure S1

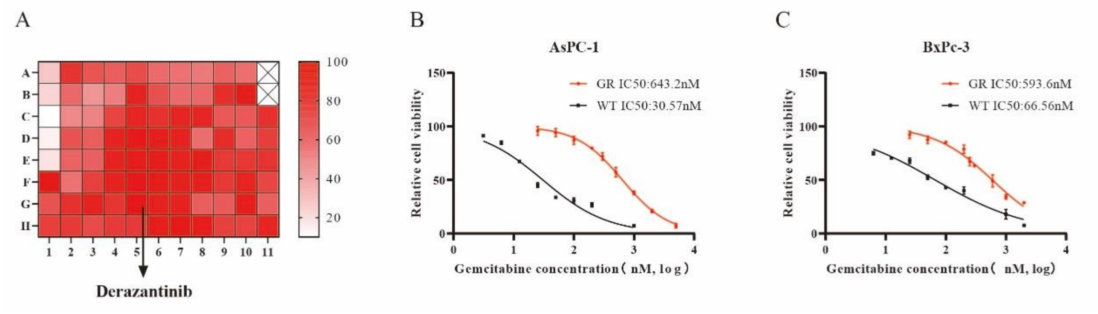

Supplementary Figure 1.

(A) Inhibitory effects of compounds at an equal concentration from the drug library on GEM-resistant PDAC cells.

(B) The IC50 values of GEM in AsPC-1(GR) GEM-resistant and AsPC-1(WT) wild-type cells.

(C) The IC50 values of GEM in BxPC-3(GR) GEM-resistant and BxPC-3(WT) wild-type cells.

Table S1. The arrangement of 86 FDA-approved Small molecule drugs in the drug library

	1	2	3	4	5	6	7	8	9	10	11
A	PX-12	Sunitinib Malate	Erlotinib	Gemcitabine	Irinotecan hydrochloride trihydrate	Chloropyramine hydrochloride	Irinotecan Hydrochloride	Gimeracil	Ifosfamide	Olaparib	
B	Cisplatin	Erlotinib hydrochloride	Genistein	Pentamidine isethionate	Abemaciclib	Silmitasertib	Pimasertib	Irinotecan	BI 2536	Abemaciclib methanesulfonate	
C	Ibrutinib	Momelotinib	Cabozantinib S-malate	Regorafenib	Cabozantinib	AVN-944	LY2090314	Vismodegib	Trametinib	Masitinib	Riviciclib hydrochloride
D	MK-0752	PND-1186	Infigratinib	Tacedinaline	Napabucasin	Tivantinib	Ivosidenib	Evofosfamide	Rabusertib	Tipifarnib	Sunitinib
E	PF-4136309	Erdafitinib	Salirasib	Gemcitabine hydrochloride	Fisogatinib	Opaganib	Oleandrin	Isoquercetin	Samotolisib	2-Deoxy-D-glucose	Exatecan Mesylate
F	Amcasertib	Talabostat mesylate	Acelarin	Devimistat	Nirogacestat	Resminostat hydrochloride	Pyrimidine	Pazopanib Hydrochloride	Dovitinib lactate	Ravuconazole	Relacorilant
G	Ciprofloxacin hydrochloride monohydrate	Trametinib (DMSO solvate)	Eniluracil	Pemigatinib	Derazantinib	CB-103	Rucaparib monocamsylate	Metarrestin	Etomidate hydrochloride	RX-3117	Coenzyme Q10
H	AZD5305	LY2880070	Hydroxychloroquine	Endovion	DM4	AMP-945	Nastorazepide	δ-Tocotrienol	Dolastatin 10	Copanlisib	Folinic acid calcium
